# Co-infection of distinct papillomavirus types in a captive North American porcupine

**DOI:** 10.1186/s12985-023-01972-w

**Published:** 2023-01-19

**Authors:** Bert Vanmechelen, Jennifer Lahoreau, Philippine Dendauw, Alexandra Nicolier, Piet Maes

**Affiliations:** 1 Department of Microbiology, Immunology and Transplantation, Laboratory of Clinical and Epidemiological Virology, Rega Institute for Medical Research, KU Leuven, Herestraat 49 Box 1040, 3000 Leuven, Belgium; 2 Parc Animalier de Sainte-Croix, 57810 Rhodes, France; 3 VetDiagnostics, Avenue de la Victoire 3, 69260 Charbonnières-Les-Bains, France

**Keywords:** North American porcupine, Erethizon dorsatum, Papillomavirus, Co-infection

## Abstract

**Background:**

Only two cases of papillomavirus infections in North American porcupines (*Erethizon dorsatum*) have been described thus far, and molecular investigation linked these cases to two distinct papillomavirus species.

**Methods:**

In this report, we present the clinical, histological and molecular investigation of a third case of a porcupine papillomavirus infection. Papillomatous lesions occurred on the upper and lower lip of an otherwise healthy three-year old female that was kept in captivity. Within one month, the lesions progressed into exophytic black nodules, followed by a temporary stabilization and ultimately spontaneous regression within seven months of their initial observation. PCR-based screening using specific primers for Erethizon dorsatum papillomavirus 1 and 2 revealed the presence of both these virus types, after which nanopore sequencing was used to determine the complete sequences of the two virus genomes.

**Results:**

One of the genomes shares 99.9% similarity with the only known sequence for Erethizon dorsatum papillomavirus 1, while the second represents a distinct lineage of Erethizon dorsatum papillomavirus 2, sharing only 93.3% similarity with the previously discovered strain.

**Conclusions:**

This report marks the first observation of a papillomavirus co-infection in a North American porcupine, although the individual contribution of the two virus types to the clinical presentation was not assessed.

## Background

The family *Papillomaviridae* contains a large collection of small, double-stranded DNA viruses that infect a diverse range of mammals, reptilians, birds and fish [1]. All papillomavirus genomes are circular, ranging from 5.7 to almost 9 kb in size, and can be divided into an early and a late region based on the temporal expression pattern of the genes they encode [1, 2]. In humans, the diversity of papillomaviruses is well studied, with hundreds of known types that can be further classified based on their pathogenic potential [3, 4]. For most animal papillomaviruses, conversely, only limited information is available, especially for those infecting non-domesticated animals. Nonetheless, the number of known animal papillomavirus types and hosts is growing continuously [5]. Most of these newly discovered viruses cluster together with other papillomaviruses found in the same host species, in line with what is known about the shaping of the papillomavirus family tree through a long history of co-evolution of virus and host [6]. However, in several cases, divergent viruses from separate clades have been identified in the same host species, hinting at the occurrence of ancient host-jumping events [7, 8]. An example of this are Erethizon dorsatum papillomavirus 1 and 2 (EdPV-1/-2), which were both discovered in North American porcupines [9–11]. While EdPV-2 clusters directly adjacent to the rodent-borne papillomaviruses of the genus *Pipapillomavirus*, albeit potentially as a separate genus, EdPV-1 is the sole member of the distantly related genus *Sigmapapillomavirus*, clustering next to the nupapillomavirus human papillomavirus 41. Despite their divergent evolutionary origin, both EdPV-1 and -2 were reported to cause wart-like papillomatous lesions.

Papillomavirus infections occur primarily in the epithelium and often resolve autonomously after a subclinical phase, although in some cases papillomavirus infections persist and evolve into warts or benign or malignant precancerous lesions [12]. The clinical severity of papillomavirus infections is determined both by the presence of environmental co-factors and the type of papillomavirus causing the infection [13]. Lesions can sometimes also be characterized by the presence of multiple virus types, although the clinical impact of such co-infections remains poorly understood. Papillomavirus co-infections have been reported in several host species, especially in the last decade, but an incomplete understanding of papillomavirus diversity and an associated lack of appropriate diagnostic tools likely results in their true prevalence being significantly underestimated [14–20]. Here, we report the first case of a papillomavirus co-infection in a North American porcupine. Using nanopore sequencing, two complete genomes could be retrieved from the same lesion, indicating the presence of both EdPV-1 and -2.

## Methods

### Sample collection

Two biopsies were collected from papillomatous lesions on the snout of a North American porcupine that was held in captivity in the ‘Parc Animalier de Sainte-Croix’, Rhodes, France. One sample was stored in buffered formalin for later histological analysis, while the second was immediately frozen at − 20 °C. From this latter biopsy, DNA was extracted using the QIAamp DNA Mini Kit (Qiagen, Hilden, Germany), according to the manufacturer’s instructions.

### Papillomavirus screening

PCR screening for the presence of papillomavirus was done using the OneStep RT-PCR kit (Qiagen), according to the manufacturer’s instructions but omitting the initial reverse transcription step. In a first attempt, five sets of degenerate primer pairs previously developed by other groups were used to screen for papillomavirus DNA: FAP59 and FAP64, AR-L1F1 and AR-L1R3, AR-L1F11 and AR-L1R10, AR-E1F2 and AR-E1R3, and AR-E1F14 and AR-E1R12 [21–23]. Because all primer sets failed to amplify the target sequence, two specific primer sets were designed based on the FAP59 and FAP64 primer pair, replacing all degenerate sites by their specific counterparts in the EdPV-1/-2 genome. Amplicons obtained using these novel sets were purified using ExoSAP-IT (Thermo Fisher Scientific, Waltham, MA, US) and sent to Macrogen Europe for sanger sequencing. The resulting chromatograms were inspected using Chromas v2.6.2. A list of all used primer sequences is provided in Table 1.

**Table 1:** Overview of used primer sequences

Primer name	Sequence (5’-3’)	References
FAP59	TAACWGTNGGNCAYCCWTATT	Forslund et al. [23]
FAP64	CCWATATCWVHCATNTCNCCATC	
AR-L1F1	TTDCAGATGGCNGTNTGGCT	Rector et al. [22]
AR-L1R3	CATRTCHCCATCYTCWAT	
AR-E1F2	ATGGTNCAGTGGGCNTATGA	Rector et al. [22]
AR-E1R3	TTNCCWSTATTNGGNGGNCC	
AR-L1F11	GGDGAYATGATGGAHATWGG	Khalafalla et al. [21]
AR-L1R10	CCATTRTTCATDCCCTGDGC	
AR-E1F14	CTTTGACACAYAYCTCAGAAAY	Khalafalla et al. [21]
AR-E1R12	AGVTCTAANCGYYCCCATARCCTT	
FAP59-EdPV-1	TGACTGTCGGTCATCCTTATT	
FAP64-EdPV-1	TATGTCCACCATGTCCGAATC	
FAP59-EdPV-2	GCACTGTTGGACATCCATATT	
FAP64-EdPV-2	CCTATATCAAACATGTCTCCATC	
EdPV-1-LT-F	AAACCCAGCTCATCATTGTAGGG	
EdPV-1-LT-R	CAACAAAGACGCTCAGTTTCTGC	
EdPV-2-LT-F	TGTGTCCTTTGACCCTAAACAGG	
EdPV-2-LT-R	GTAGCTTCCACACAAGACGTTCC	

### Whole genome sequencing

Based on the obtained partial L1 sequences, two primer sets were designed, one for each genome, aimed at amplifying the remainder of the genome in a single amplicon (Table 1). Amplification was done using LongAmp Taq DNA Polymerase (New England Biolabs, Ipswich, MA, US) according to the manufacturer’s instructions. Following an initial three-minute incubation at 94 °C, the DNA fragments were amplified using forty cycles of twenty seconds at 94 °C, forty-five seconds at 59 °C, and six minutes and fifteen seconds (EdPV-1) or seven minutes (EdPV-2) at 65 °C, followed by a final elongation step of ten minutes at 65 °C. The resulting amplicons were pooled and purified by a 1 × AMPure XP bead purification with two 70% ethanol washes. The purified DNA was prepared for nanopore sequencing using the SQK-RAD004 kit (Oxford Nanopore Technologies, Oxford, UK) according to the manufacturer’s instructions. The resulting library was loaded on a FLO-FLG001 flow cell and sequenced for 21 h.

### Data analysis

Basecalling was performed using the super-accurate model of Guppy v5.0.11. Blastn was used to subset the reads by comparing them with the EdPV-1 and -2 reference sequences (GenBank: AY684126 and MH376689), after which each subset was de novo assembled using Canu v2.0 [24]. Medaka v1.4.3 was used to polish the resulting contigs, which had a minimum coverage of 1,000X at each position. The final contigs were joined with the previously obtained partial L1 sequences using SeqMan v7.0.0, yielding two complete, circular genome sequences. The finished genomes were manually inspected for any residual errors using CLC Genomics Workbench v20.0.2. The complete genome sequences were submitted to GenBank under the accession numbers MZ647948 and MZ647949. Open reading frame and feature annotation was done using PuMA [25]. For the comparison of the two EdPV-2 sequences, an alignment was made using the built-in Muscle algorithm of MEGA7, which was then used for visual comparison using SimPlot v3.5.1, employing a window size of 200 bp and a step size of 20 bp [26–28].

## Results

### Clinical presentation

**Fig. 1:**
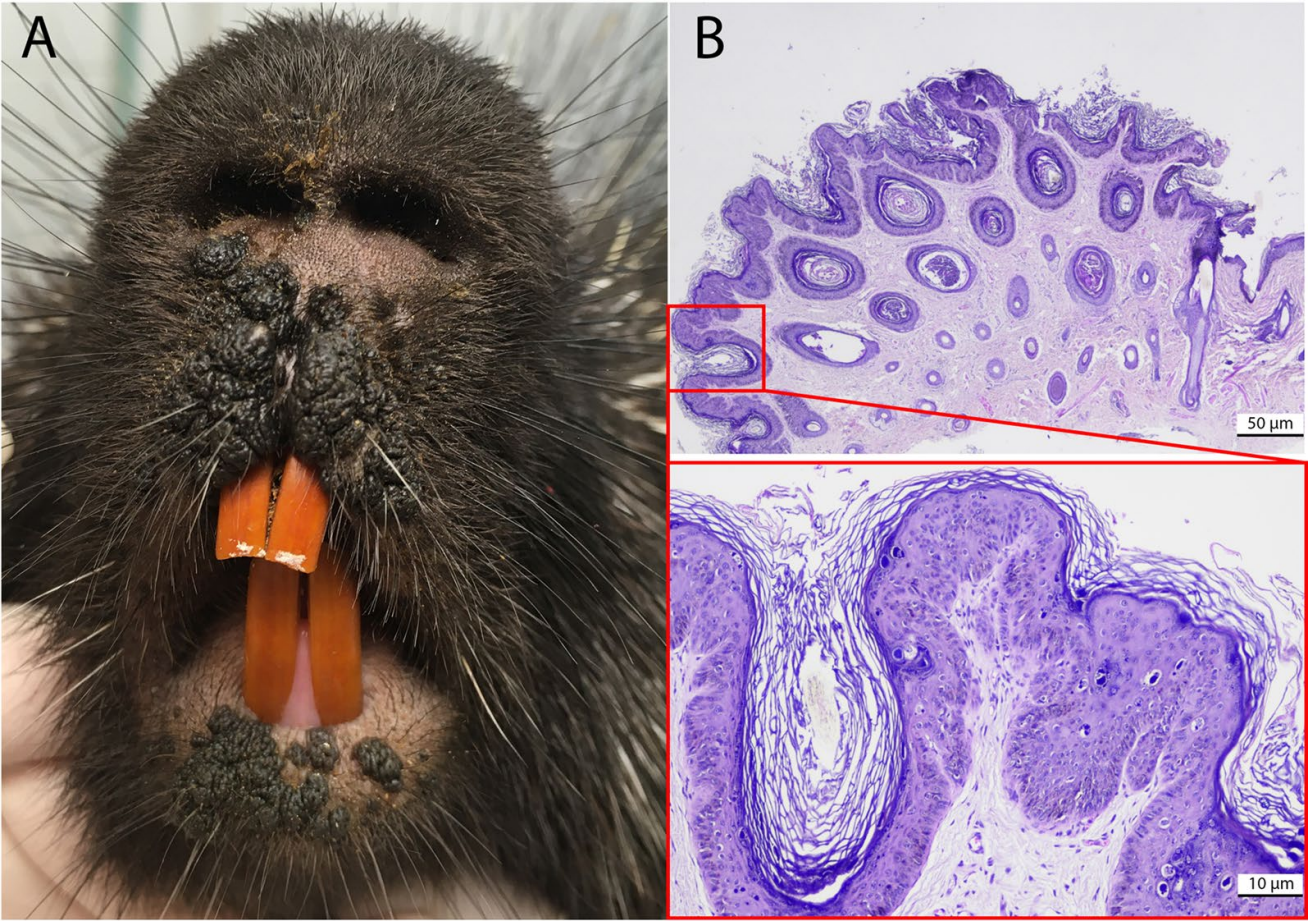
Anatomical and histological appearance of the papillomatous lesions. **A** Lesions manifested themselves as multiple exophytic black nodules on the inferior and superior lips. **B** Histology using a hematoxylin and eosin staining (original magnification 2× and 10x) showed papillary epidermal hyperplasia associated with moderate basket-wave hyperkeratosis, hypergranulosis and diffuse pigmentation of the epidermis

Lesions resembling a putative papillomavirus infection were observed on the muzzle of an otherwise healthy, three-year old female North American porcupine, kept in captivity at the ‘Parc Animalier de Sainte-Croix’, Rhodes, France. The lesions were first observed on May 26th, at which point they were described as “little spots on the nose”. Four weeks later, on June 23rd, a more detailed investigation was performed under general anesthesia. By then, the lesions had evolved into multiple exophytic black nodules on the inferior and superior lips, slightly more on the right side (Fig. 1). Two biopsies were taken at this time, for histological and molecular analysis. Histological examination showed a focally extensive moderate papillary epidermal hyperplasia sharply demarcated from the normal epidermis, associated with moderate basket-wave hyperkeratosis, multifocal slight vacuolar degeneration of keratinocytes, marked hypergranulosis with large keratohyaline granules and marked hyperplasia of basal melanocytes. The dermis was slightly infiltrated by pleomorphic inflammatory cells. The lesions remained stable until the end of September, after which they started to regress spontaneously. By November, only a few nodules remained and by December, the lesions had resolved completely. A thirteen-year old male porcupine that was housed together with the affected female remained unaffected.

### Molecular investigation

Because the lesions resembled those of a papillomavirus infection (see [10]), DNA extract of one of the biopsies was screened for the presence of papillomavirus DNA using several previously published degenerate primer pairs targeting the conserved L1 or E1 regions of the genome (Table 1). However, all these screening assays failed to amplify part of a papillomavirus genome. To further rule out a papillomavirus as the causative agent, two new, specific primer pairs were designed by replacing all degenerate positions in the FAP59-FAP64 set by their exact counterparts from the EdPV-1 and -2 reference genomes, the only two papillomaviruses previously reported in North American porcupines. Intriguingly, both these pairs managed to amplify part of a papillomavirus genome, and sanger sequencing of the resulting amplicons confirmed the presence of both EdPV-1 and EdPV-2. To further characterize these two viruses, the obtained amplicon sequences were used as templates for the design of two long-template PCRs that spanned the remainder of the circular genomes, with estimated fragment sizes of 7.2 and 8.7 kb. Nanopore sequencing of these two fragments and subsequent read assembly, contig polishing and joining with the original amplicons resulted in the generation of two complete virus genome sequences.

**Fig. 2:**
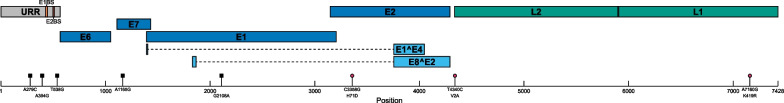
Genome organization of the detected EdPV-1 strain. The virus strain described here differs only in eight positions from the reference sequence (GenBank: AY684126). All differences are highlighted, with circles indicating non-synonymous mutations. Open reading frame and feature annotation was done using PuMA [25]. URR = untranslated regulatory region, E1BS = E1 binding site, E2BS = E2 binding site

**Fig. 3:**
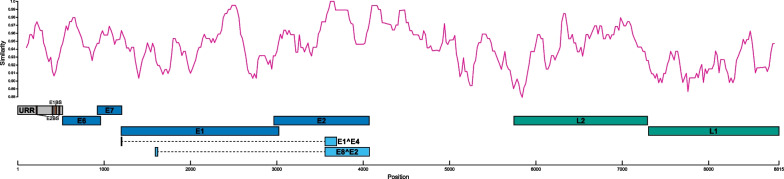
Genome organization of the detected EdPV-2 strain. A comparison of the EdPV-2 genome described here with the reference sequence (GenBank: MH376689) shows a roughly even distribution of dissimilarities throughout the genome. Comparison made using SimPlot V3.5.1 [28]. Open reading frame and feature annotation was done using PuMA [25]. URR = untranslated regulatory region, E1BS = E1 binding site, E2BS = E2 binding site

The EdPV-1 genome is nearly identical to the previously published EdPV-1 genome sequence (GenBank: AY684126), having the exact same length (7,428 nucleotides) and sharing 99.89% sequence identity (Fig. 2). The two genomes differ in only eight positions, three of which are located in the upstream regulatory region (URR). Of the remaining mutations, three are synonymous, resulting in a single amino acid change in the E2 (H71D), L2 (V2A) and L1 (K419R) proteins. The EdPV-2 genome is markedly more dissimilar from its published counterpart (GenBank: MH376689), sharing only 93.33% nucleotide identity. While the genome presented here is also slightly longer, having a length of 8,815 nucleotides, the two genomes are nonetheless sufficiently similar to consider them two variants of the same papillomavirus type. As shown in Fig. 3, the differences between the two sequences are spread roughly evenly throughout the genome. Intriguingly, the secondary non-coding region between the early and late regions is as equally conserved as the rest of the genome. In the case of EdPV-2, this region is exceptionally large, although its precise function remains to be elucidated. We have previously published a detailed overview of the known regulatory motives in the genome of EdPV-2 [9]. Nearly all of these motives are identical to the ones found in the genome presented here, with only minor differences in the E2 DNA-binding motif (**GR**A**N**A**LKC**W**R**H**R**) and the L2 arginine/lysine-rich region (**KRRRRRR** vs **KRRRRRRR**).

## Discussion

This report represents only the third description of a papillomavirus infection in a North American porcupine. While the two previous cases, reported in 2005 and 2018, were caused by two distinct viruses belonging to different papillomavirus genera, the case presented here appears to have been a mixed infection, caused by variants of the two previously reported Erethizon dorsatum papillomaviruses [9–11]. However, while both EdPV-1 and EdPV-2 were detected, it is unclear to which extent both viruses contributed to the clinical presentation of the papillomatous lesions. It is possible that co-infection with two different viruses had a cumulative effect, aggravating the progression of the disease. Conversely, it might also be that only one of the two viruses played a causal role in the clinical presentation, and that the detection of the second virus was no more than an accidental observation. Morphologically, the lesions seen here resembled those previously observed for EdPV-2 [10]. For EdPV-1, a comparison is more difficult because there are no images available of the only known EdPV-1 case. However, based on the provided description of the lesions as “*multiple white to light brown lobulated, raised, firm masses on the foot pads and facial skin, ranging in size from 2 to 10 mm*” it is possible that this case actually resembled the early stage of the infection described here [11]. In the case described here, the lesions regressed spontaneously without any therapeutic intervention and disappeared completely after 6–7 months. Because no serial sampling was performed, it is unclear at which stage of the infection the viral presence of either strain started to reduce and if this decline occurred at similar rates for both viruses. Although unfortunately not performed for the case described here, such serial sampling through non-invasive skin or mucosal swabs might help monitor the evolution of viral persistence in future comparable cases, providing new insights into the kinetics of animal papillomavirus infections. Likewise, the swabbing of seemingly uninfected co-housed animals could be performed to screen for subclinical infections that might otherwise be overlooked.

Co-infections by different papillomavirus types have been described previously for different animal species, in some cases by more than two types at the same time [14–20]. In 2016, Daudt and colleagues even reported the finding of seven distinct bovine papillomavirus types in the same lesion [19]. In their report, they note that co-infections are likely more prevalent than currently known, partly because PCR-based methods might fail to detect certain types. Using several commonly used PCR screening assays, we also failed to detect papillomavirus DNA, even though we had previously successfully used two of these assays for the detection of EdPV-2 [9]. However, despite belonging to the same type, there are notable differences between this EdPV-2 variant and the previously described one, including several mutations in the primer binding sites of the aforementioned assays. Using more specific primers, we were able to successfully amplify both EdPV-1 and -2 genome fragments, but, while unlikely, it cannot be ruled out that other additional papillomavirus types might have been overlooked.

The EdPV-2 genome presented here differs significantly from the previously reported sequence. Given the low evolutionary rate of papillomaviruses, this finding clearly indicates the circulation of distinct EdPV-2 lineages [6]. Further research is needed to determine whether these lineages can be found ubiquitously, or if they are confined to specific geographic regions or *Erethizon dorsatum* subspecies. Interestingly, both genomes are marked by the presence of an exceptionally large secondary non-coding region between the early and late regions. Even though this region appears mostly void of known regulatory motives, its conservation in distinct lineages nonetheless hints at a functional relevance. Unlike the EdPV-2 genome, the EdPV-1 genome presented here differs by only 0.1% from the only other known EdPV-1 sequence, despite having been found more than fifteen years apart and in animals from different continents [11]. However, because both EdPV-1-infected porcupines were kept in captivity, it is plausible that there was direct contact between the ancestors of these animals or those of their cage mates in the not too distant past. Further research will be necessary to elucidate if also for EdPV-1 there exist distinct lineages and whether there are other porcupine-specific papillomavirus lineages or types that remain to be discovered.

## Data Availability

Genome sequences generated in this study are available via their GenBank accession numbers MZ647948 and MZ647949.
